# Integrating machine learning and nontargeted plasma lipidomics to explore lipid characteristics of premetabolic syndrome and metabolic syndrome

**DOI:** 10.3389/fendo.2024.1335269

**Published:** 2024-03-15

**Authors:** Xinfeng Huang, Qing He, Haiping Hu, Huanhuan Shi, Xiaoyang Zhang, Youqiong Xu

**Affiliations:** ^1^ The Affiliated Fuzhou Center for Disease Control and Prevention of Fujian Medical University, Fuzhou, China; ^2^ School of Public Health, Fujian Medical University, Fuzhou, China

**Keywords:** machine learning, nontargeted lipidomics, premetabolic syndrome, metabolic syndrome, biomarkers

## Abstract

**Objective:**

To identify plasma lipid characteristics associated with premetabolic syndrome (pre-MetS) and metabolic syndrome (MetS) and provide biomarkers through machine learning methods.

**Methods:**

Plasma lipidomics profiling was conducted using samples from healthy individuals, pre-MetS patients, and MetS patients. Orthogonal partial least squares-discriminant analysis (OPLS-DA) models were employed to identify dysregulated lipids in the comparative groups. Biomarkers were selected using support vector machine recursive feature elimination (SVM-RFE), random forest (rf), and least absolute shrinkage and selection operator (LASSO) regression, and the performance of two biomarker panels was compared across five machine learning models.

**Results:**

In the OPLS-DA models, 50 and 89 lipid metabolites were associated with pre-MetS and MetS patients, respectively. Further machine learning identified two sets of plasma metabolites composed of PS(38:3), DG(16:0/18:1), and TG(16:0/14:1/22:6), TG(16:0/18:2/20:4), and TG(14:0/18:2/18:3), which were used as biomarkers for the pre-MetS and MetS discrimination models in this study.

**Conclusion:**

In the initial lipidomics analysis of pre-MetS and MetS, we identified relevant lipid features primarily linked to insulin resistance in key biochemical pathways. Biomarker panels composed of lipidomics components can reflect metabolic changes across different stages of MetS, offering valuable insights for the differential diagnosis of pre-MetS and MetS.

## 1 Background

MetS comprises a cluster of “cardiometabolic risk” factors, including high blood sugar, hypertension, hypertriglyceridemia, low high-density lipoprotein cholesterol, and abdominal obesity. Pre-MetS denotes a set of clinical and biochemical features manifesting metabolic irregularities in specific aspects, albeit not fully meeting the diagnostic criteria for MetS (1–4). The combined impact of these components and ongoing metabolic disruptions significantly increase the risk of cardiovascular disease (CVD) (3) and cancer (4). According to previous research (5), the risk of CVD in pre-MetS is 1.5 to 2.3 times higher than that in individuals without MetS components, while MetS increases the risk by 3.44 to 4.42 times.

As of now, the most widely accepted diagnostic criteria for MetS include those established by the National Cholesterol Education Program Adult Treatment Panel III (NCEP ATP III) (6), the International Diabetes Federation (IDF) (7), and the Joint Commission of the China Adult Dyslipidemia Control Guide (JCDCG) (8) in China. Among these, the IDF criteria stipulate abdominal obesity as a prerequisite, while the JCDCG criteria incorporate postprandial blood glucose into the definition of hyperglycemia. Furthermore, the revised ATP III criteria enhance screening for individuals at high risk by lowering the diagnostic threshold for fasting blood glucose to 5.6 mmol/L. Compared to other criteria, the revised ATP III criteria are more straightforward and efficient, offering advantages in capturing individuals with metabolic abnormalities in large-scale community screening. Global prevalence rates for MetS (IDF criteria) and pre-MetS (IDF criteria) were reported to be 16.46% and 14.72%, respectively (9). Prior investigations indicated that the incidence of MetS stabilized after the age of 46 (10), and the contribution of each metabolic factor associated with MetS was not equal (5). A cross-sectional study showed that the most common risk factors for pre-MetS and MetS are hypertension and abdominal obesity (11), while another small-scale study revealed a higher prevalence of high triglycerides and hypertension (12). A recent cohort study assessed the relative contributions of four major MetS risk factors in a large population, ranked from highest to lowest as high blood sugar, hypertension, dyslipidemia, and obesity (13). Metabolic phenotypes observed in MetS patients with hyperglycemia are similar to those with all four risk factors, indicating that individuals with hyperglycemia and hypertension are more predisposed to developing cardiovascular and cerebrovascular diseases.

Many studies combined machine learning with lifestyle-related and anthropometric features to detect and prevent MetS (11), yet the mechanisms underlying the development of MetS remain incompletely understood (6). However, research suggests that insulin resistance, disturbances in glucose and lipid metabolism, and chronic inflammation interact through multiple signaling mechanisms, with abnormal lipid metabolism being a common denominator (14, 15). The clustered metabolic disruptions in MetS lead to worsening lipid metabolism abnormalities, eventually culminating in significant cardiovascular disease. Thus, apart from clinical markers, lipidomics is employed to discover diagnostic and prognostic biomarkers associated with MetS, enhancing our understanding of its etiology. For instance, a Dutch study found that approximately 100 lipids, mainly triglycerides, were positively correlated with MetS, while 10 lipids were negatively correlated (16).

Given the escalating global prevalence of MetS, early identification of at-risk individuals and predicting patient responses to treatment is vital. The development of novel biomarkers for MetS has potential for use in diagnosis and treatment of this disorder. Researchers have extensively screened population and clinical features for predicting MetS (17) and identifying related factors (18). However, no study has deeply investigated changes in lipid metabolites across different physiological states of pre-MetS and MetS. Thus, gaining a deeper understanding of lipid changes could aid in establishing monitoring programs for pre-MetS and MetS, ultimately reducing the incidence of cardiovascular disease. This study aims to construct optimal pre-MetS and MetS identification models through a combination of machine learning techniques and nontargeted lipidomics, contributing to preventive health care in the population.

## 2 Materials and methods

### 2.1 Study design and participants

Between March 2021 and June 2021, a multistage stratified cluster random sampling method was used to select residents undergoing routine health check-ups from 18 villages in 6 towns in Jin’an District, Fuzhou City. A preliminary survey was conducted with a response rate of 95.75%, involving 1,800 permanent residents who had lived in the area for at least 6 months. The inclusion criteria were as follows: (1) age ≥ 18 years; and (2) exclusion of individuals with coronary heart disease, myocardial infarction, angina pectoris, stroke, malignancy, chronic obstructive pulmonary disease, chronic urinary system diseases (e.g., stones, prostatitis, chronic nephritis), or missing baseline data. A total of 8,715 individuals met these criteria. 28 MetS patients were enrolled and matched 1:1 and 2:1 by sex and age with pre-MetS and normal individuals, respectively, resulting in a final study cohort of 70 participants.

### 2.2 Variable definitions and survey content

MetS diagnosis followed the revised ATP III (6), where participants were defined as having MetS if they had any three of the following five phenotypes: (1) systolic blood pressure (SBP) ≥ 130 mmHg and/or diastolic blood pressure (DBP) ≥ 85 mmHg; (2) triglycerides (TG) ≥ 1.7 mmol/L; (3) fasting plasma glucose (FPG) ≥ 5.6 mmol/L; (4) high-density lipoprotein cholesterol (HDL-C) < 1.03 mmol/L for men or < 1.29 mmol/L for women; and (5) abdominal obesity defined as waist circumference (WC) ≥ 90 cm for men or ≥ 85 cm for women. Pre-MetS was defined as having one or two MetS components. A self-designed unified questionnaire was used to collect information on personal health status, medical history, and lifestyle behaviors (exercise, smoking, alcohol consumption, sleep). Physical examinations included height, weight, waist circumference (measured twice and averaged), and blood pressure measurements (measured thrice using UR-9000F). Laboratory biochemical tests were conducted on venous blood collected from participants in a fasting state. Serum total cholesterol (TC), low-density lipoprotein cholesterol (LDL-C), HDL-C, TG, and FBG were measured using enzymatic colorimetric methods. Serum uric acid (SUA), creatinine (Cre) and blood urea nitrogen (BUN) levels were measured using a colorimetric method on a Hitachi 7100 automatic biochemistry analyzer.

### 2.3 Nontargeted lipidomics analysis

After fasting for at least 12 hours, morning venous blood samples were collected from all participants using venipuncture, and the samples were stored at -80°C until further nontargeted lipidomics analysis. The lipidomics contents were measured at Shanghai Applied Technology Co., Ltd., China (http://www.aptbiotech.com/). The project utilizes a nontargeted lipidomics analysis platform based on the UPLC-Orbitrap mass spectrometry system from China New Life Technology Co., Ltd. Lipid identification and data preprocessing are carried out using LipidSearch software by Thermo Scientific™.

Preparation of quality control (QC) samples involves combining equal amounts of samples from each group to create the QC mixture. QC samples serve not only to assess instrument status and chromatography−mass spectrometry system equilibration before injection but also to evaluate the overall experimental system stability.

Sample preprocessing involved thawing samples on ice, vortex-mixing, and transferring 100 μL to a 1.5 mL centrifuge tube. Subsequently, 200 μL of 4°C water was added, followed by vortex mixing. Next, 240 μL of prechilled methanol was added and mixed by vortexing, and then 800 μL of MTBE was added and mixed by vortexing. The mixture was subjected to 20 minutes of ultrasonication in a low-temperature water bath, followed by 30 minutes of room-temperature incubation. Afterward, centrifugation at 14,000 g and 10°C for 15 minutes was performed, and the upper organic phase was collected. The samples were dried using nitrogen gas and stored at -80°C.

Chromatographic separation employed the UHPLC Nexera LC-30A ultrahigh-performance liquid chromatography system. The column temperature was set at 45°C, and the flow rate was 300 μL/min. The mobile phase consisted of two components: A - 10 mM ammonium formate in acetonitrile-water solution (acetonitrile:water = 6:4, v/v) and B - 10 mM ammonium formate in acetonitrile-isopropanol solution (acetonitrile:isopropanol = 1:9, v/v). The gradient elution program was as follows: 0-2 minutes, B was held at 30%; 2-25 minutes, B linearly changed from 30% to 100%; and 25-35 minutes, B was held at 30%. Throughout the analysis, samples were kept in an autosampler at 10°C. To mitigate the impact of instrument signal fluctuations, samples are analyzed in a randomized sequence.

Mass spectrometric separation was conducted using both electrospray ionization positive and negative ion modes. After UHPLC separation, analysis was performed using a QExactive Plus mass spectrometer (Thermo Scientific™).

### 2.4 Data analysis

**Figure 1:**
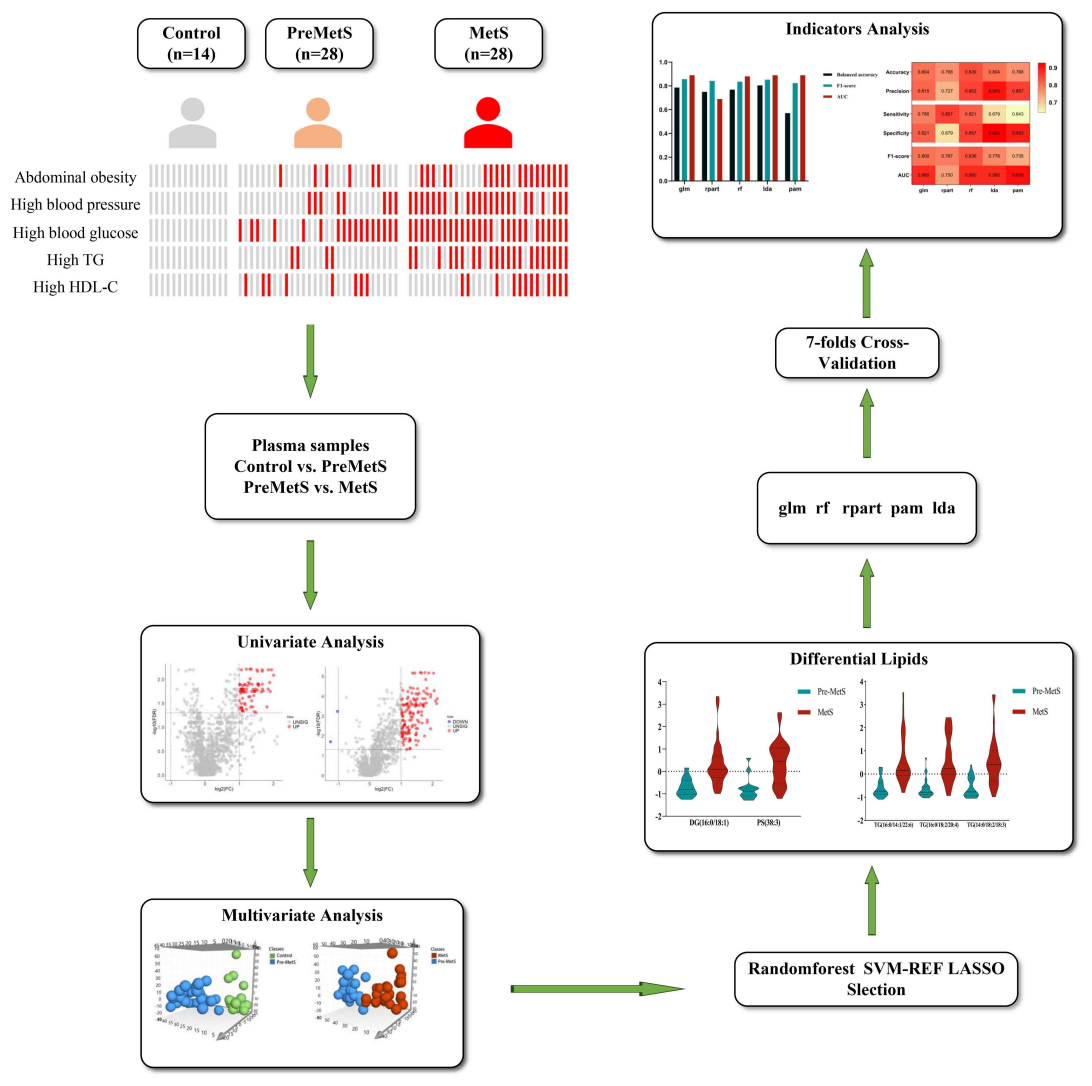
Study design and data analysis workflow.

Data were double-entered using EpiData 3.1 software, and statistical analysis was performed using SPSS 26.0 and R 4.2.2 software. For normally distributed data, the mean ± standard deviation (x̄ ± s) is used, while for non-normally distributed data, the median (upper quartile, lower quartile) is used, represented as Median (M), quartile range (P25, P75). Group differences are compared using analysis of variance (ANOVA) or non-parametric tests. Count data are presented as composition ratios and rates (n, %), and group differences are analyzed using chi-square tests. Lipid identification, peak extraction, and lipid characterization were performed using Lipid Search. Univariate analysis was conducted on the extracted data, and volcano plots were used for visualization. Prior to evaluating the predictive performance of various machine learning methods, data from each group underwent exploratory multivariate statistical analysis using seven-fold cross-validation and OPLS-DA, including normalization, logarithmic transformation, and autoscaling, to examine potential outliers or systematic variations (FDR < 0.05). The variable importance for the projection (VIP) values were used to measure the influence strength and explanatory power of each lipid molecule on sample classification discrimination in each group. Lipid molecules with VIP > 1 significantly contribute to the model interpretation. Lipid molecules with VIP > 1.5, P < 0.05, and FC > 1.5 were selected as significantly different based on the criteria. The machine learning models in this study included generalized linear model (glm), recursive partitioning and regression (rpart), random forest (rf), linear discriminant analysis (lda), and prediction analysis for microarrays (pam). Before evaluating the predictive performance of various machine learning methods, exploratory multivariate statistical data analysis using OPLS-DA was conducted on normalized, logarithmically transformed, and autoscaled data from each group to check for potential outliers or systematic changes (FDR < 0.05). The variable intersection of support vector machine recursive feature elimination (SVM-RFE), rf, and least absolute shrinkage and selection operator (LASSO) regression was applied to each pairwise comparison (control vs. pre-MetS and pre-MetS vs. MetS) to identify the most discriminative variables. After selecting variables, five machine learning models were established. Validation was performed using 7-fold cross-validation, and during model development, 10-fold cross-validation was used for training and testing to obtain optimal parameters. In the model development process, adjustments were made to the hyperparameters of each algorithm (such as cost values, kernel functions, and the number of trees in the training dataset). Therefore, using the best hyperparameters, our model was trained and tested on six folds and validated on the remaining fold, repeated seven times across the entire dataset (**Figure 1**).

## 3 Results

### 3.1 Clinical characteristics

We tested 1361 nontargeted lipid metabolites in the plasma of patients with pre-MetS or MetS. The important sociodemographic factors and laboratory tests for each participant are reported in **Table 1**. Results from ANOVA and Chi-square tests indicated no statistically significant differences (P > 0.05) in gender, age, education level, marital status, occupation type, smoking status, alcohol consumption, exercise habits, TC, LDL-C, Cre, and BUN among the groups (**Table 1**).

**Table 1:** Baseline.

Characteristics	Total (n = 70)	Normal (n = 14)	pre-MetS(n = 28)	MetS(n = 28)	*P*
Gender					1.000
Male	40 (57.1)	8 (57.1)	16 (57.1)	16 (57.1)	
Female	30 (42.9)	6 (42.9)	12 (42.9)	12 (42.9)	
Age(years)	53.61 ± 8.1	54.29 ± 10.36	52.96 ± 7.89	53.93 ± 7.29	0.856
Education					0.320
High school and above	2 (6.9)	1 (20.0)	0 (0)	1 (8.3)	
Below high school	27 (93.1)	4 (80.0)	12 (100)	11 (91.7)	
Marital status					0.799
Married/cohabiting	67 (95.7)	14 (100)	27 (96.4)	26 (92.9)	
Divorced/widowed/separated	3 (4.3)	0 (0)	1 (3.6)	2 (7.1)	
Occupations					0.337
Brain work	12 (17.1)	2 (14.3)	7 (25.0)	3 (10.7)	
Physical labor	43 (61.4)	11 (78.6)	14 (50.0)	18 (64.3)	
Retired/unemployed	15 (21.4)	1 (7.1)	7 (25.0)	7 (25.0)	
Smoking status					0.844
Never	48 (68.6)	10 (71.4)	18 (64.3)	20 (71.4)	
Smoking/quitting	22 (31.4)	4 (28.6)	10 (35.7)	8 (28.6)	
Alcohol consumption					0.199
No alcohol/moderate drinking	63 (90.0)	14 (100)	26 (92.9)	23 (82.1)	
Alcohol abuse	7 (10.0)	0 (0)	2 (7.1)	5 (17.9)	
Exercise habits					0.767
Medium to high intensity exercise	29 (41.4)	7 (50.0)	11 (39.3)	11 (39.3)	
Lack of exercise	41 (58.6)	7 (50.0)	17 (60.7)	17 (60.7)	
Sleep duration(h/d)					0.046
7-8	42 (60.0)	9 (64.3)	21 (75.0)	12 (42.9)	
<7 or >9	28 (40.0)	5 (35.7)	7 (25.0)	16 (57.1)	
BMI (kg/m^2^)	24.62 ± 3.74	23.41 ± 3.23	23.8 ± 3.66	26.05 ± 3.7	0.029
WC (cm)	83.82 ± 9.68	76.84 ± 4.53	80.22 ± 9.4	89.79 ± 7.89	< 0.001
SBP (mmHg)	127.3 (116.4, 136.2)	119.3 (115.3, 128.4)	119 (114.6, 131.5)	136.2 (127.8, 142.4)	< 0.001
DBP (mmHg)	79.77 ± 10.07	73.14 ± 8.93	76.73 ± 7.73	86.13 ± 9.32	< 0.001
FBG (mmol/L)	5.7 (5.3, 6.5)	5.3 (5.1, 5.5)	5.7 (5.2, 6.5)	6.1 (5.7, 6.7)	< 0.001
OGTT-2h(mmol/L)	11.3 (6.1, 12)	6.1 (5.2, 7.2)	8.8 (5.7, 11.5)	12 (11.4, 13.3)	< 0.001
TC (mmol/L)	5.04 ± 0.87	4.68 ± 0.52	4.93 ± 0.89	5.33 ± 0.92	0.052
TG (mmol/L)	1.4 (1.0, 1.9)	0.7 (0.6, 0.9)	1.2 (1, 1.6)	2.2 (1.7, 3.4)	< 0.001
HDL-C (mmol/L)	1.25 ± 0.28	1.43 ± 0.21	1.27 ± 0.27	1.15 ± 0.28	0.007
LDL-C (mmol/L)	3.03 ± 0.77	3.04 ± 0.44	3.02 ± 0.93	3.03 ± 0.75	0.997
SUA (μmmol/L)	356 (302.6, 421.1)	321 (234.5, 359.2)	344.5 (300, 405)	387 (329.2, 448.5)	0.017
Cre (mmol/L)	69.65 ± 12.74	67.26 ± 16.03	68.39 ± 10.89	72.1 ± 12.71	0.412
BUN (mmol/L)	4.94 ± 1.28	4.81 ± 1.07	4.87 ± 1.19	5.08 ± 1.47	0.762

Data are presented as the means ± SDs or frequencies (percentages).

MetS metabolic syndrome, SD standard deviation, WC waist circumference, SBP systolic blood pressure, DBP diastolic blood pressure, BMI body mass index, FPG fasting plasma glucose, OGTT-2h Oral Glucose Tolerance Test - 2 hours, TC total cholesterol, TG triglycerides, HDL-C high-density lipoprotein cholesterol, LDL-C low-density lipoprotein cholesterol, Cre creatinine, BUN blood urea nitrogen.

*P* < 0.05 was considered statistically significant.

### 3.2 Identification of differentially expressed lipids

**Figure 2:**
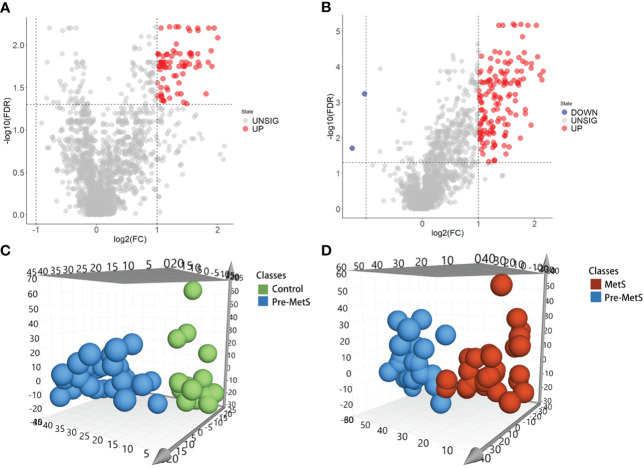
Identification of lipids related to pre-MetS and MetS. **(A)** Volcano plot of candidate lipid metabolism biomarkers in the pre-MetS group. **(B)** Volcano plot of candidate lipid metabolism biomarkers in the MetS group. **(C)** Orthogonal Partial Least Squares Discriminant Analysis (OPLS-DS) score plot between the pre-MetS and Normal groups. **(D)** OPLS-DS score plot between the MetS and pre-MetS groups. Lipid metabolites colored by their chemical categories. Multivariate analysis was conducted using a seven-fold cross-validation method.

To investigate the role of lipids in the pathogenesis of pre-MetS and MetS, we performed subsequent analysis using the expression profiles of nontargeted lipidomics from the plasma of pre-MetS patients compared to healthy controls and MetS patients. Differential expression analysis of the 1,361 lipid expression profiles revealed that there were 77 significantly upregulated lipids in pre-MetS patients compared to healthy controls and 141 significantly upregulated lipids in pre-MetS patients compared to MetS patients. Additionally, there were 2 significantly downregulated lipids in pre-MetS patients compared to MetS patients (**Figures 2A, B**). VIP values were calculated for each metabolite through the OPLS-DA model, and metabolites with VIP values > 1.5 were considered the most important. The number of latent variables in the OPLS-DA model was chosen based on sevenfold cross-validation. OPLS-DA score plots demonstrated separation between pre-MetS patients and healthy controls, as well as between pre-MetS patients and MetS patients (**Figures 2C, D**). The cumulative R2Y values from the OPLS-DA model were 0.709 and 0.589, and the cumulative Q2 values were 0.453 and 0.342 for the pre-MetS vs. control and pre-MetS vs. MetS comparisons, respectively. From the 1,361 candidate metabolites, 50 and 89 metabolites were selected as candidates based on VIP > 1.5, FDR < 0.05, and log_2_|FC| > 1 (**Supplementary Tables 1**, **2**).

### 3.3 Feature selection using LASSO, rf and SVM-RFE

**Figure 3:**
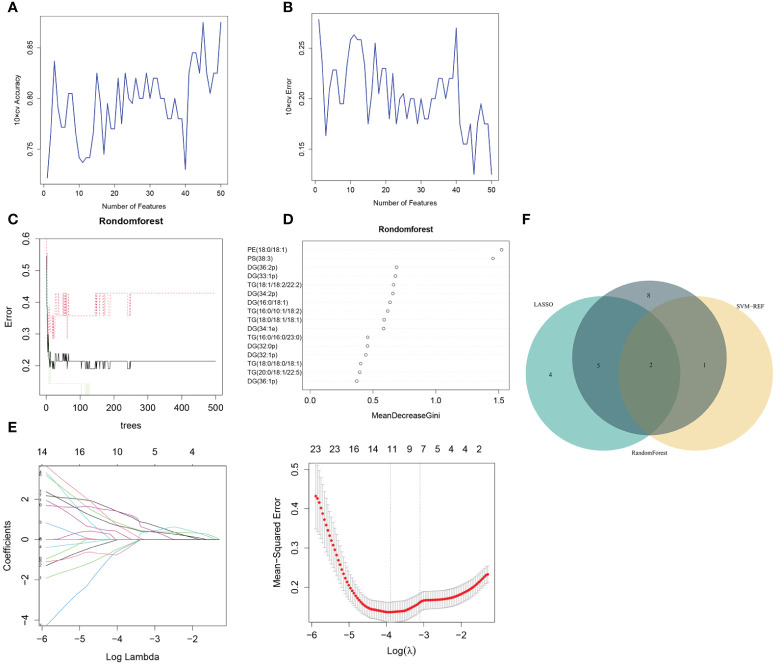
Pre-MetS lipid feature selection. **(A, B)** Biomarker signature lipid expression validation via SVM–RFE algorithm selection. **(C)** Random forest error rate versus the number of classification trees. **(D)** The top 16 relatively important lipids. **(E)** Adjustment of feature selection in the LASSO model. **(F)** Three algorithmic Venn diagrams screening lipids. All three algorithms employed ten-fold cross-validation for feature selection.

Three algorithms—LASSO, rf and SVM-RFE—were employed to select the core lipid features associated with pre-MetS patients. For SVM-RFE, to prevent overfitting, when including three features, PE(18:0/18:1), PS(38:3), and DG(16:0/18:1), the classifier accuracy reached a maximum value, and the error was minimized (**Figures 3A, B**). Using rf, 15 lipids were identified with relative importance >0.4, including: PE(18:0/18:1), PS(38:3), DG(36:2p), DG(33:1p), TG(18:1/18:2/22:2), DG(34:2p), DG(16:0/18:1), TG(16:0/10:1/18:2), TG(18:0/18:1/18:1), DG(34:1e), TG(16:0/16:0/23:0), DG(32:0p), DG(32:1p), and TG(18:0/18:0/18:1) (**Figures 3C, D**).

Regarding the LASSO algorithm, after tenfold cross-validation, the optimal lambda (λ) was 0.02038657. Using a λ value of 0.045 that corresponded to the minimum partial likelihood deviance (**Figure 3E**), 11 feature lipids were selected: TG(18:1/18:2/22:2), PS(38:3), DG(16:0/18:1), TG(20:0/18:1/22:5), DG(36:1p), TG(16:0/16:0/16:0), DG(34:2p), TG(16:0/16:0/17:0), TG(25:0/18:1/18:1), DG(34:2p), and TG(16:0/18:1/20:3). Two lipids with shared features were identified from the LASSO, rf, and SVM-RFE algorithms: PS(38:3) and DG(16:0/18:1) (**Figure 3F** and **Table 2**).

**Table 2:** The situation of two significantly different lipid metabolites identified by three machine learning methods in plasma between Normal and Pre-MetS.

Molecule	Subclass	Formula	m/z	RT (min)	VIP	Log2|FC|	*P*	FDR
PS (38:3)-H	PS	C44 H79 O10 N1 P1	812.54	12.23	2.11	1.20	1.79E-05	0.01
DG (16:0/18:1)+NH4	DG	C37 H74 O5 N1	612.56	13.00	2.17	1.35	1.79E-05	0.01

The number before the ratio in parentheses is the length of the carbon chain, the number after the ratio is the number of double bonds on the carbon chain; -H and +NH4 are lipid molecule change groups. m/z: Mass-to-Charge Ratio; RT (min): Retention Time; VIP: Variable Importance in Projection; Log2|FC|: Log2 Fold Change; P: P-value; FDR: False Discovery Rate.

### 3.4 Feature selection using LASSO, rf and SVM-RFE for MetS patients

**Figure 4:**
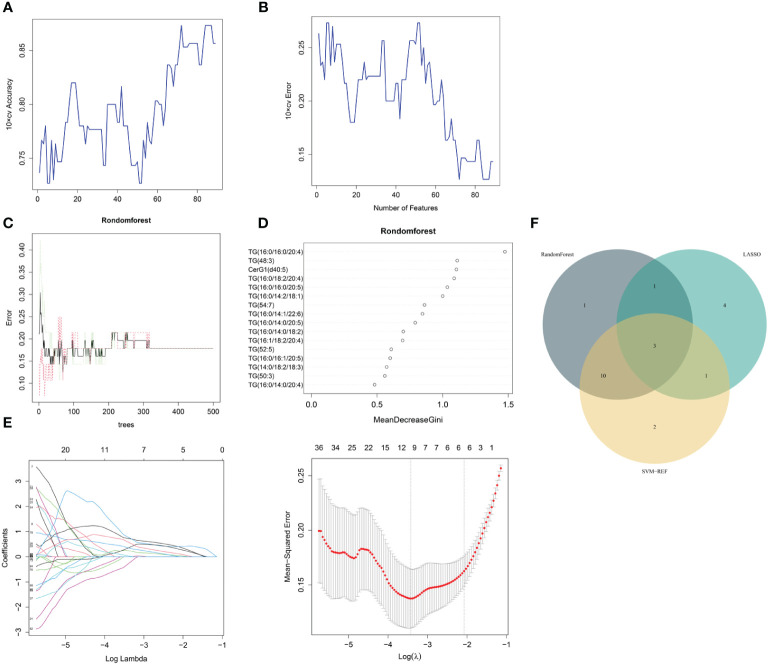
MetS lipid feature selection. **(A, B)** Biomarker signature lipid expression validation via SVM–RFE algorithm selection. **(C)** Random forest error rate versus the number of classification trees. **(D)** The top 17 relatively important lipids. **(E)** Adjustment of feature selection in the minimum absolute shrinkage and selection operator model (LASSO). **(F)** Three algorithmic Venn diagrams screening lipids. All three algorithms employed ten-fold cross-validation for feature selection.

The same three algorithms (LASSO, rf and SVM-RFE) were utilized to select the core lipid features associated with MetS patients. For SVM-RFE, when including 17 features, TG(52:5), TG(16:0/16:0/20:5), TG(16:0/14:0/20:5), TG(16:0/18:2/20:4), CerG1(d40:5), DG(32:0), TG(16:0/10:1/18:2), TG(15:0/16:1/17:0), TG(16:0/14:0/18:2), TG (48:3), TG (16:0/14:2/18:1), TG (50:3), TG (16:0/16:0/20:4), TG (16:1/18:2/18:3), TG (16:0/16:1/20:5), TG (16:0/14:1/22:6), and TG(16:1/18:2/20:4), the classifier accuracy reached a maximum value, and the error was minimized (**Figures 4A, B**). Using rf, 16 lipids were identified with relative importance >0.4, including: TG(16:0/16:0/20:4), TG(48:3), CerG1(d40:5), TG(16:0/18:2/20:4), TG (16:0/16:0/20:5), TG(16:0/14:2/18:1), TG(54:7), TG(16:0/14:1/22:6), TG (16:0/14:0/20:5), TG(16:0/14:0/18:2), TG(16:1/18:2/20:4), TG(52:5), TG (16:0/16:1/20:5), TG(14:0/18:2/18:3), TG(50:3), and TG(16:0/14:0/20:4) (**Figures 4C, D**). Regarding the LASSO algorithm, after tenfold cross-validation, the optimal lambda was 0.033. Using a λ value of 0.126 that corresponded to the minimum partial likelihood deviance (**Figure 4E**), 9 feature lipids were selected: TG (16:0/16:0/16:0), DG (18:2/20:4), TG (14:0/18:2/18:3), PI (16:0/16:1), TG (16:0/18:2/20:4), TG (18:1/18:2/22:5), TG (16:0/14:1/22:6), DG (32:0p), and DG (30:1p).

Three shared feature lipids were identified from the LASSO, rf and SVM-RFE algorithms: TG (16:0/14:1/22:6), TG (16:0/18:2/20:4), and TG (14:0/18:2/18:3) (**Figure 4F** and **Table 3**).

**Table 3:** The situation of three significantly different lipid metabolites identified by three machine learning methods in plasma between Pre-MetS and MetS.

Molecule	Subclass	Formula	m/z	RT (min)	VIP	Log2|FC|	*P*	FDR
TG (16:0/14:1/22:6)+NH4	TG	C55 H96 O6 N1	866.72	17.82	1.96	1.64	3.54E-08	6.88E-06
TG (16:0/18:2/20:4)+NH4	TG	C57 H102 O6 N1	896.77	19.53	2.12	1.43	2.11E-08	6.88E-06
TG (14:0/18:2/18:3)+NH4	TG	C53 H96 O6 N1	842.72	17.63	1.9	1.81	3.82E-07	4.00E-05

The number before the ratio in parentheses is the length of the carbon chain, the number after the ratio is the number of double bonds on the carbon chain; three sets of numbers indicate that the compound consists of three longer carbon chains; +NH4 are lipid molecule change groups. m/z: Mass-to-Charge Ratio; RT (min): Retention Time; VIP: Variable Importance in Projection; Log2|FC|: Log2 Fold Change; P: P-value; FDR: False Discovery Rate.

### 3.5 Machine learning models for pre-MetS and MetS identification

**Figure 5:**
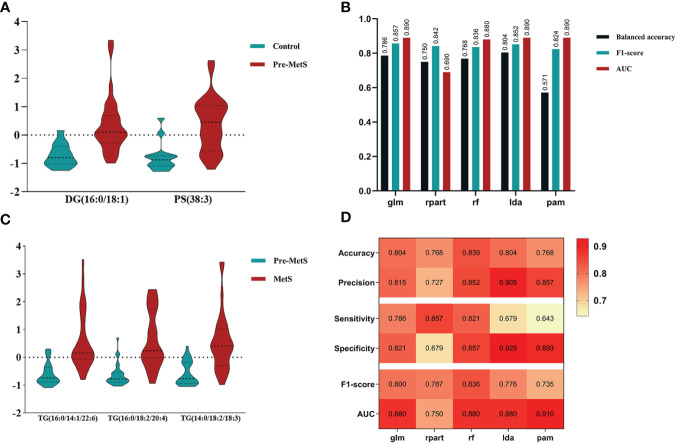
**(A)** Determining lipid panels in pre-MetS based on three variable-selection methods. **(B)** Performance evaluation metrics for each ML-based model distinguish control individuals from pre-MetS patients. **(C)** Determining lipid panels in MetS based on three variable-selection methods. **(D)** Performance evaluation metrics for each ML-based model distinguishing pre-MetS from MetS. From left to right: glm, rpart, rf, lda, and pam. The repeated ten-fold cross-validation was used for model performance validation, while the ten-fold cross-validation was utilized for model training and parameter tuning.

**Figure 6:**
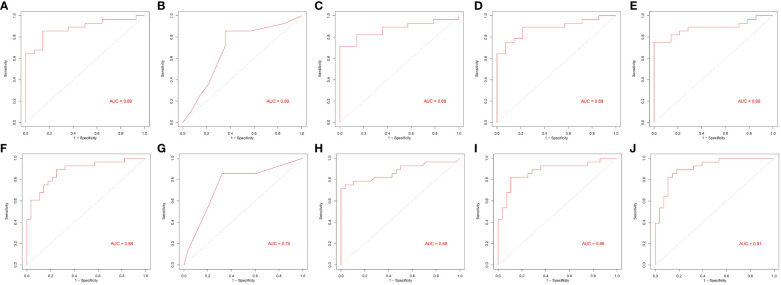
Area under the receiver operating characteristic curves of five machine learning algorithms. **(A–E)** and **(F–J)** From left to right: generalized linear model (glm), recursive partitioning and regression (rpart), random forest (rf), linear discriminant analysis (lda), and prediction analysis for microarrays (pam).

An important application of lipidomics is the identification of potential disease biomarkers. Based on the feature selection results, PS(38:3) and DG(16:0/18:1) were identified as two important lipids for identifying pre-MetS (**Figure 5A**). We compared the performance of five popular machine learning algorithms on the test dataset to determine the optimal classification method for lipidomics data. These algorithms included glm, rpart, rf, lda, and pam. Due to the imbalanced sample sizes between the pre-MetS and control groups, we used balanced accuracy, F1-score, and AUC to evaluate the models. Among them, lda was identified as the best model with the highest balanced accuracy and F1-score, all exceeding 0.8 (**Figure 5B** and **Figures 6A–E**).

Based on the feature selection results, TG(16:0/14:1/22:6), TG(16:0/18:2/20:4), and TG(14:0/18:2/18:3) were identified as three important lipids for identifying MetS (**Figure 5C**). We used six performance metrics to evaluate the models, and rf demonstrated the best performance, with all metrics exceeding 0.8 (**Figure 5D** and **Figures 6F–J**).

## 4 Discussion

Metabolic risk factors present significant global challenges, necessitating effective strategies for early intervention. In this study, which involved a small sample of pre-MetS and MetS patients, we screened differential lipids between the two groups based on the expression levels of 1361 lipids and established identification models. Our results revealed significant differences in the levels of 77 lipids for pre-MetS compared to the control group and 143 lipids for MetS compared to the control group (**Figure 2**). Furthermore, through machine learning, we selected the optimal lipid panel and models for identifying pre-MetS and MetS (**Figures 3**, **4**), achieving model evaluation metrics exceeding 0.8 (**Figure 5**). Previous studies have mainly focused on identifying metabolites associated with MetS (16, 17). In contrast, our research emphasizes using machine learning-based lipid selection for identifying pre-MetS and MetS patients, particularly targeting middle-aged and elderly individuals at risk of metabolic dysfunction, and promoting effective interventions to modify risk factors, rather than relying solely on traditional risk factors.

Our study differs from others in that we explored the differences in lipid metabolites between pre-MetS and MetS for the first time. Several explanations support this research. First, considering the complexity and heterogeneity of pre-MetS and MetS components (19), a comprehensive assessment of lipid metabolism may better reflect the underlying disease progression, providing fundamental insights into the dynamic changes of MetS and enabling more specific treatments for patients. Second, considering the cumbersome nature of physical examinations during widespread screening and the potential for significant measurement errors and reduced efficiency due to variations in instruments, the diagnosis of pre-MetS and MetS may lead to false-positives. Therefore, lipid metabolites could serve as useful auxiliary indicators. In contrast to traditional classification, this study classified participants into three groups: control, pre-MetS, and MetS, aiming for a large-scale community-based screening program for MetS and cardiovascular disease prevention. In our research, the combinations of two and three biomarkers corresponded to LDA and rf models, respectively, with both exhibiting good discriminative ability in the validation set through sevenfold cross-validation (AUC of 0.89 for pre-MetS vs. control and 0.88 for pre-MetS vs. MetS) (**Figures 6D, H**).

We found that higher levels of plasma DGs and TGs were positively correlated with the risk of pre-MetS and MetS. Consistent with previous studies (16), we identified DG(36:2) as associated with MetS through OPLS-DA and univariate analysis (**Supplementary Table 1**). Conversely, while previous research found that DG(34:1) was associated with MetS, we found it to be associated with pre-MetS. This is not surprising, as DGs act as bioactive lipids, serving as second messengers in insulin resistance induction, and TGs play a critical role in regulating fatty acid oxidation and lipid synthesis (20), and are widely used to predict cardiovascular risk (21).

We identified a class of phospholipids (PE(18:0/18:1), PE(18:0/20:5), PS(38:3)) positively correlated with pre-MetS. Phosphatidylserine (PS) is involved in cell membrane composition and various signaling pathways, providing signals for immune cell recognition and phagocytosis during cell apoptosis (22). Interestingly, immune-related dysregulation has been found to play a prominent role in pre-MetS (12), which might be due to the biochemical pathways differing in the heterogeneity of pre-MetS populations in our study compared to other studies. We also found that levels of ceramides (Cer(d40:4), Cer(d40:5), Cer(d42:4)) were positively correlated with MetS. Total ceramide content is positively correlated with insulin resistance (23). In fact, ceramides are involved in inducing cell apoptosis through various downstream targets (24) and are associated with atherosclerosis (25).

Our study achieved favorable screening results with a relatively small number of lipids combined with corresponding models, yielding an AUC > 0.8. This indicated that the lipids we identified serve as excellent screening tools. However, the study has some limitations. Firstly, it is an exploratory study with a small sample size, which may lead to a certain degree of overfitting, although we mitigated this issue through various machine learning methods. Secondly, the LC-MS lipidomics technique can only differentiate lipids based on identification algorithms for subion, parent ion, and neutral loss scans, rather than providing clear and unique identification (26). This complicates pathway enrichment analysis of different lipids in the study. Lastly, the participants in this study were all residents from coastal areas of China, and the results may not be extrapolated to other countries and inland regions. We hope that future research, combining larger sample sizes and multiomics studies, will further explore these findings.

## 5 Conclusion

In this initial lipidomics analysis of pre-MetS and MetS, we identified relevant lipid features and selected 50 and 89 plasma lipid metabolites associated with pre-MetS and MetS patients, respectively. Furthermore, through machine learning, we selected two sets of plasma metabolites composed of PS(38:3), DG(16:0/18:1), and TG(16:0/14:1/22:6), TG(16:0/18:2/20:4), TG(14:0/18:2/18:3) as biomarkers for the identification models of pre-MetS and MetS in this study. Our results indicate that the identified biomarkers can reflect metabolic changes at different stages of MetS, providing a new perspective for monitoring disease progression and treatment response in pre-MetS and MetS patients. These findings hold promise for the differential diagnosis of pre-MetS and MetS, laying a foundation for future diagnostics and treatments.

## Data availability statement

The datasets presented in this study can be found in online repositories. The names of the repository/repositories and accession number(s) can be found in the article/**Supplementary Material**.

## Ethics statement

The studies involving humans were approved by ethical review committee of Fuzhou Center for Disease Control and Prevention. The studies were conducted in accordance with the local legislation and institutional requirements. The participants provided their written informed consent to participate in this study. Written informed consent was obtained from the individual(s) for the publication of any potentially identifiable images or data included in this article.

## Author contributions

HH: Formal analysis, Validation, Writing – review & editing. XH: Formal analysis, Writing – original draft. HS: Investigation, Writing – review & editing. QH: Data curation, Writing - review & editing. XZ: Conceptualization, Methodology, Writing – review & editing, Funding acquisition, Resources. YX: Conceptualization, Methodology, Writing – review & editing.
